# Cardiac magnetic resonance identifies raised left ventricular filling pressure: prognostic implications

**DOI:** 10.1093/eurheartj/ehac207

**Published:** 2022-05-04

**Authors:** Pankaj Garg, Rebecca Gosling, Peter Swoboda, Rachel Jones, Alexander Rothman, Jim M Wild, David G Kiely, Robin Condliffe, Samer Alabed, Andrew J Swift

**Affiliations:** Department of Infection, Immunity and Cardiovascular Disease, The University of Sheffield, Sheffield, UK; Norwich Medical School, University of East Anglia, Norwich, UK; Norfolk and Norwich University Hospitals NHS Foundation Trust, Norwich, UK; Department of Infection, Immunity and Cardiovascular Disease, The University of Sheffield, Sheffield, UK; The Institute of Cardiovascular and Metabolic Medicine, University of Leeds, UK; Department of Infection, Immunity and Cardiovascular Disease, The University of Sheffield, Sheffield, UK; Department of Infection, Immunity and Cardiovascular Disease, The University of Sheffield, Sheffield, UK; Department of Infection, Immunity and Cardiovascular Disease, The University of Sheffield, Sheffield, UK; Department of Infection, Immunity and Cardiovascular Disease, The University of Sheffield, Sheffield, UK; Sheffield Pulmonary Vascular Disease Unit, Sheffield Teaching Hospitals NHS Foundation Trust, Sheffield, UK; Sheffield Pulmonary Vascular Disease Unit, Sheffield Teaching Hospitals NHS Foundation Trust, Sheffield, UK; Department of Infection, Immunity and Cardiovascular Disease, The University of Sheffield, Sheffield, UK; Department of Infection, Immunity and Cardiovascular Disease, The University of Sheffield, Sheffield, UK

**Keywords:** Left ventricular filling pressure, Right heart catheterization, Cardiovascular magnetic resonance

## Abstract

**Aims:**

Non-invasive imaging is routinely used to estimate left ventricular (LV) filling pressure (LVFP) in heart failure (HF). Cardiovascular magnetic resonance (CMR) is emerging as an important imaging tool for sub-phenotyping HF. However, currently, LVFP cannot be estimated from CMR. This study sought to investigate (i) if CMR can estimate LVFP in patients with suspected HF and (ii) if CMR-modelled LVFP has prognostic power.

**Methods and results:**

Suspected HF patients underwent right heart catheterization (RHC), CMR and transthoracic echocardiography (TTE) (validation cohort only) within 24 h of each other. Right heart catheterization measured pulmonary capillary wedge pressure (PCWP) was used as a reference for LVFP. At follow-up, death was considered as the primary endpoint. We enrolled 835 patients (mean age: 65 ± 13 years, 40% male). In the derivation cohort (*n* = 708, 85%), two CMR metrics were associated with RHC PCWP:LV mass and left atrial volume. When applied to the validation cohort (*n* = 127, 15%), the correlation coefficient between RHC PCWP and CMR-modelled PCWP was 0.55 (95% confidence interval: 0.41–0.66, *P* < 0.0001). Cardiovascular magnetic resonance-modelled PCWP was superior to TTE in classifying patients as normal or raised filling pressures (76 vs. 25%). Cardiovascular magnetic resonance-modelled PCWP was associated with an increased risk of death (hazard ratio: 1.77, *P* < 0.001*).* At Kaplan–Meier analysis, CMR-modelled PCWP was comparable to RHC PCWP (≥15 mmHg) to predict survival at 7-year follow-up (35 vs. 37%, χ^2^ = 0.41, *P*  = 0.52).

**Conclusion:**

A physiological CMR model can estimate LVFP in patients with suspected HF. In addition, CMR-modelled LVFP has a prognostic role.

## Structured Graphical Abstract

**Structured Graphical Abstract:**
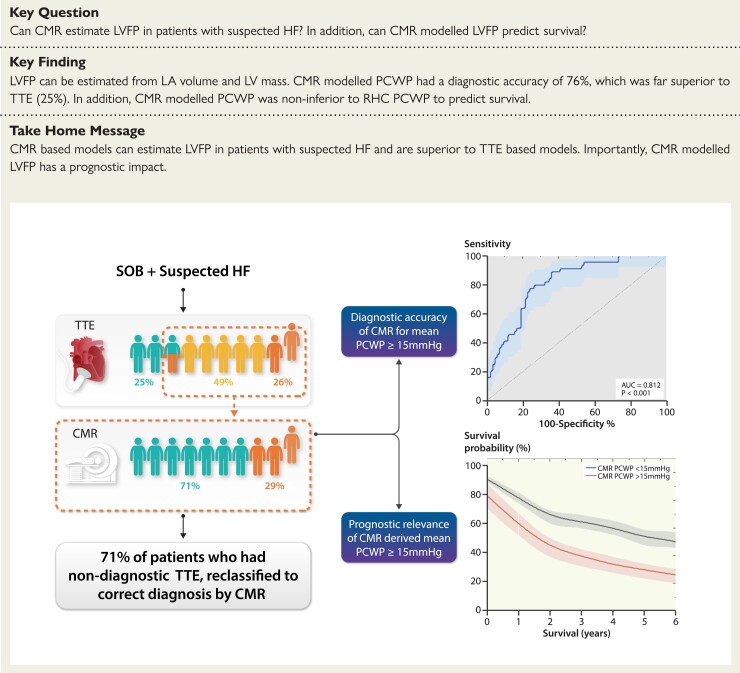
Patients presenting with breathlessness underwent TTE, CMR, and RHC. Cardiovascular magnetic resonance-modelled PCWP and TTE-estimated PCWP were both compared with invasively measured PCWP. The diagnostic accuracy of our CMR model was 71% compared with 25% for TTE. In cases where TTE was non-diagnostic (indeterminate or incorrect diagnosis), CMR correctly reclassified the correct diagnosis in 71%. Furthermore, CMR-derived PCWP was an independent predictor of survival. *CMR, cardiac magnetic resonance; HF, heart failure; PCWP, pulmonary capillary wedge pressure; SOB, shortness of breath; TTE, transthoracic echocardiography; RHC, right heart catheterization*.

**See the editorial comment for this article ‘Cardiovascular magnetic resonance for the assessment of left ventricular filling pressure in heart failure’, by Anna Baritussio and Vivek Muthurangu, https://doi.org/10.1093/eurheartj/ehac247.**

## Introduction

Heart failure (HF) presents a significant social and economic burden and it is on the rise.^1^ The underlying pathophysiology of HF is raised intracardiac filling pressures. Identification of raised left ventricular filling pressure (LVFP) is the cornerstone of HF diagnosis.^2^ Reference methods for LVFP assessment are invasive catheter-based methods. In routine clinical practice, right heart catheterization (RHC) is preferred for comprehensive invasive evaluation of cardiovascular haemodynamics. In the absence of lesions in the pulmonary venules, veins, left atrium, and mitral valve, the pulmonary capillary wedge pressure (PCWP), obtained by occluding the pulmonary artery, provides an accurate measurement of LVFP.^3^ Elevated PCWP is not only used to establish the diagnosis of HF^4^ but also identifies patients at an increased risk of death^5,6^ and lowering PCWP reduces HF hospitalizations.^7^

At a population level, the invasive strategy is not feasible to diagnose and monitor treatment progress in patients with HF. Hence, non-invasive methods are preferred and as such, transthoracic echocardiography (TTE) is the mainstay of initial LVFP assessment.^8^ Cardiac magnetic resonance (CMR) imaging has emerged as an important imaging tool for clarification of aetiology of HF and further sub-phenotyping.^9,10^ The main benefit of CMR is its enhanced precision in functional and volumetric assessment.^11^ Currently, there is no CMR model available that predicts LVFP. It also remains unclear if such a CMR model will offer any prognostic advantage.

Thus, we carried out this study to (i) investigate whether CMR functional and geometric parameters are associated with invasively measured PCWP in patients with suspected or proven HF; (ii) develop a CMR model to predict PCWP; and (iii) investigate if the CMR-modelled PCWP may be used for risk stratification of patients.

## Methods

### Study population

**Figure 1:**
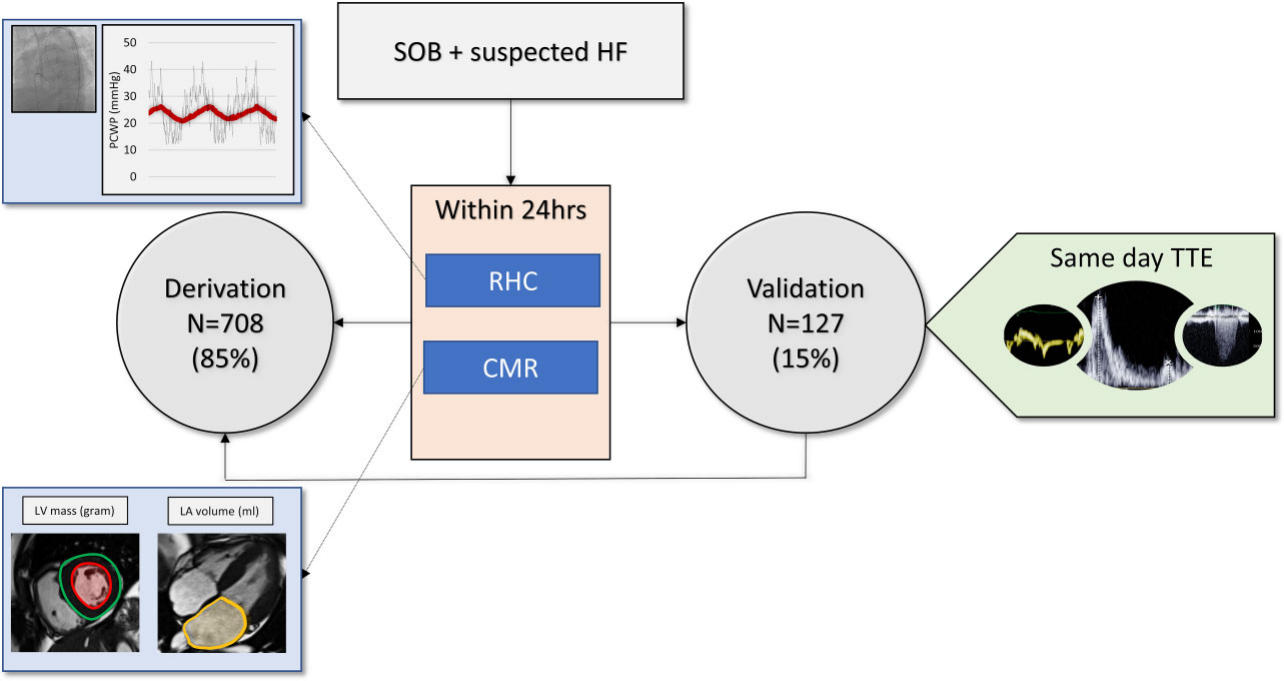
Study protocol. This study included patients who were referred to our centre over a 8-year period (2012–2020) for further assessment of breathlessness. Right heart catheterization and cardiac magnetic resonance were performed within 24 h in all cases. Eight hundred and thirty-five patients were included and allocated to derivation (85%) and validation (15%) cohorts. In the validation cohort, we obtained transthoracic echocardiography results within 24 h for comparison with cardiac magnetic resonance. CMR, cardiac magnetic resonance; HF, heart failure; LA, left atrial; LV, left ventricular; PCWP, pulmonary capillary wedge pressure; RHC, right heart catheterization; SOB, shortness of breath; TTE, transthoracic echocardiography.

This study included patients who were referred to our centre over a 8-year period (2012–2020) for further assessment of breathlessness. This population includes patients from several databases. Right heart catheterization and CMR were performed within 24 h in all cases (*Figure 1*). The mean follow-up period was 4 ± 2 years. Inclusion criteria included signs or symptoms of HF, age >18 years, and informed consent. Exclusion criteria included pulmonary arterial hypertension (Type 1), contraindications to RHC or CMR, including claustrophobia and end-stage HF. This study was approved by the Sheffield Teaching Hospitals and approved by the National Research Ethics Service (16/YH/0352) in the UK. The study complied with the Declaration of Helsinki.

### Invasive study

Right heart catheterization was performed using a balloon-tipped 7.5 French thermodilution catheter (Becton-Dickinson, Franklin Lakes, New Jersey). The PCWP was recorded using standard techniques and averaged over several cardiac cycles instead of the end-expiratory method which can overestimate it^12^ (*Figure 1*). It was recorded when patients were relaxed with a minimal beat-to-beat variation. Cardiac output was measured using the thermodilution technique.

### Cardiac magnetic resonance study

Cardiac magnetic resonance was performed using a 1.5 T whole-body GE HDx scanner (GE Healthcare, Milwaukee, USA) with an eight-channel cardiac coil. Four-, two-, three-chamber and short-axis cine images were acquired using a retrospectively cardiac-gated multi-slice steady-state free precession sequence (TR 2.8 ms, TE 1.0 ms, flip angle 50°, field of view 48 × 43.2, 256 × 256 matrix, 125 kHz bandwidth, and slice thickness 8–10 mm) in keeping with standard protocols.^13^ A GE Advantage Workstation 4.1 was used for offline image analysis by an investigator, blinded to all clinical and RHC data. Manual contouring of the endocardial and epicardial surfaces, excluding the papillary muscles, was performed on the stack of short-axis cine images to obtain left ventricular (LV) end-diastolic volume (LVEDV), LV end-systolic volume (LVESV), right ventricular (RV) end-diastolic volume (RVEDV) and RV end-systolic volume (RVESV). From end-diastolic and end-systolic volumes, LV stroke volume (LVSV), LV ejection fraction (LVEF), RV stroke volume (RVSV) and RV ejection fraction (RVEF) were calculated. Cardiac magnetic resonance-derived LV cardiac output was calculated by multiplying LVSV by the heart rate. Ventricular mass was calculated at end-diastole; the interventricular septum was considered part of the left ventricle. The left atrial endocardium was contoured in the four- and two-chamber views to obtain maximum left atrial volume (LAV) just before the mitral valve opening (LV end-systolic phase) using the biplane area–length method. The haemodynamic variations between RHC and CMR were checked by comparing the cardiac output of both modalities.

### Transthoracic echocardiography study

Clinically indicated TTE was performed according to local practice guidelines within 24 h of RHC. Multiple echocardiographic parameters were measured in keeping with the British Society of Echocardiography (BSE) minimum data set.^14^ Left atrial pressure was estimated from TTE using the American Society of Echocardiography (ASE) algorithm which classifies patients as normal, raised or indeterminate left atrial pressure based upon mitral inflow parameters, tissue Doppler imaging, tricuspid regurgitation velocity and LAV index.^8^

### Statistical analysis

All clinically acquired data were treated as normally distributed. Continuous variables were presented as mean ± standard deviation. Categorical data were reported as frequencies and percentages. A two-sample independent *t*-test was used to compare continuous variables. The χ^2^ test was used for categorical data. Paired *t*-test was used to compare cardiac outputs by CMR and RHC. The data were split into derivation (85% *n* = 706) and validation cohorts (15% *n* = 127). From the derivation cohort, univariate linear regression was used to generate Pearson correlation coefficients for individual CMR metrics compared with PCWP by RHC and multivariate regression was used to develop a model relating several CMR metrics. Supervised machine learning penalized regression models were also tested for CMR PCWP model. The final model was applied to the validation cohort and receiver operating characteristic analysis was performed to assess the diagnostic performance of CMR PCWP to detect raised RHC PCWP. Kaplan–Meier analysis and Cox proportional hazard model were used for multivariate analysis of prognosis. Statistical analysis was performed in SPSS version 22 (IBM, Chicago, IL, USA) and confirmed in MedCalc (MedCalc Software, Ostend, Belgium version 19.1.5). Supervised machine learning penalized regression was undertaken in StataIC 16. Unless otherwise stated, all statistical tests were two-tailed, and a *P*-value of <0.05 was deemed significant.

## Results

### Study population

In total, 835 patients were included in the study. Of these, 521 (62%) had a normal PCWP (<15 mmHg) and 314 (38%) had a raised PCWP (≥15 mmHg), as measured by RHC. The patient characteristics are summarized in *Table 1*. Of the whole population, 337 (40%) were male and the mean age was 65 years. The primary diagnosis identified as the cause of the patient’s breathlessness was left heart disease in 497 (60%) patients, lung disease in 160 (19%) patients and pulmonary hypertension in 178 (21%) patients. Of those with left heart disease, 442 (89%) had HF with preserved ejection fraction (HFpEF) and 55 (11%) had HF with reduced ejection fraction (HFrEF).

**Table 1:** Patient characteristics, cardiac haemodynamic data, and cardiac magnetic resonance data stratified by right heart catheterization pulmonary capillary wedge pressure

	RHC PCWP <15 mmHg (*n* = 521)	RHC PCWP ≥15 mmHg (*n* = 314)	*P*-value
Age (years)	64.2 ± 13.9	69.6 ± 10.9	<0.0001
Male sex	221 (42%)	116 (37%)	0.1271
Body surface area (m^2^)	1.88 ± 0.2	1.92 ± 0.35	0.0315
HFpEF	219 (42%)	223 (71%)	<0.0001
HFmrEF	21 (4%)	11 (3.5%)	0.7009
HFrEF	14 (2.7%)	9 (2.9%)	0.8784
Other	267 (51.2%)	71 (22.6%)	<0.0001
Cardiac haemodynamic data			
Heart rate (bpm)	77.2 ± 14.0	73.6 ± 14.4	0.0004
Systolic blood pressure (mmHg)	140.1 ± 24.7	v	<0.0001
Diastolic blood pressure (mmHg)	77.4 ± 12.2	78.3 ± 13.7	0.3192
Mean arterial pressure (mmHg)	101.1 ± 16.7	104.3 ± 18.9	0.012
Mean PCWP (mmHg)	10.1 ± 2.9	20.2 ± 5.1	<0.0001
Mean right atrial pressure (mmHg)	7.6 ± 4.6	13.8 ± 5.2	<0.0001
Mean pulmonary artery pressure (mmHg)	35.4 ± 14.4	42.2 ± 11.6	<0.0001
Systolic pulmonary artery pressure (mmHg)	58.9 ± 24.9	68.5 ± 21.4	<0.0001
Diastolic pulmonary artery pressure (mmHg)	20.0 ± 10.0	24.9 ± 8.3	<0.0001
Arterial oxygen saturations (%)	94.4 ± 4.0	93.9 ± 4.5	0.0969
Venous oxygen saturations (%)	66.5 ± 8.2	64.9 ± 8.9	0.0087
Cardiac output (L)	5.0 ± 2.0	5.1 ± 1.8	0.7903
Cardiac index (L/min/m^2^)	2.7 ± 1.1	2.7 ± 0.89	0.9211
CMR data			
Left ventricular end-diastolic volume (mL)	103.0 ± 31.8	121.7 ± 42.4	<0.0001
Left ventricular end-systolic volume (mL)	34.4 ± 16.9	42.5 ± 26.7	<0.0001
Left ventricular stroke volume (mL)	68.6 ± 22.7	79.5 ± 25.9	<0.0001
Left ventricular ejection fraction (%)	67.0 ± 10.9	66.5 ± 11.9	0.5322
Left ventricular mass (g)	93.3 ± 30.1	105.7 ± 38.0	<0.0001
Right ventricular end-diastolic volume (mL)	140.4 ± 60.8	159.5 ± 62.2	<0.0001
Right ventricular end-systolic volume (mL)	82.2 ± 49.7	90.1 ± 47.9	0.024
Right ventricular stroke volume (mL)	58.2 ± 24.4	69.4 ± 28.6	<0.0001
Right ventricular ejection fraction (%)	43.7 ± 13.9	45.2 ± 13.1	0.1365
Left atrial volume (cm^3^)	62.1 ± 28.3	104.4 ± 51.0	<0.0001
Left ventricular end-diastolic volume (mL) (indexed)	54.8 ± 15.5	63.7 ± 21.5	<0.0001
Left ventricular end-systolic volume (mL) (indexed)	18.2 ± 8.6	22.2 ± 13.9	<0.0001
Left ventricular stroke volume (mL) (indexed)	36.6 ± 11.5	41.6 ± 13.2	<0.0001
Left ventricular ejection fraction (%) (indexed)	49.3 ± 13.3	55.0 ± 18.7	<0.0001
Left ventricular mass (g) (indexed)	74.7 ± 30.4	83.5 ± 32.0	0.0001
Right ventricular end-diastolic volume (mL) (indexed)	43.5 ± 25.2	47.2 ± 25.0	0.0433
Right ventricular end-systolic volume (mL) (indexed)	31.1 ± 12.8	36.3 ± 14.5	<0.0001

RHC, right heart catheterization; PCWP, pulmonary capillary wedge pressure; HFpEF, heart failure with preserved ejection fraction; HFmrEF, heart failure with mid-range ejection fraction; HFrEF, heart failure with reduced ejection fraction.

Patients with a raised PCWP had a higher prevalence of HFpEF (71%) than patients with a normal PCWP (42%). Systolic blood pressure was higher in patients with a raised PCWP (150.0 ± 29.3) compared with patients with a normal PCWP (140.1 ± 24.7 mmHg) (*P* < 0.0001). Right atrial pressure was also higher in those with a raised PCWP (raised PCWP: 14 ± 5 mmHg, normal PCWP: 8 ± 5 mmHg, *P* < 0.0001). The cardiac index was comparable between the two groups (*P* = 0.92).

### Cardiac magnetic resonance evaluation

Standard CMR metrics were evaluated to provide a volumetric assessment (*Table 1*). Patients with a raised PCWP were found to have higher LVEDV and LVESV (122 ± 42 mL and 43 ± 27 mL, respectively, *P* < 0.0001). Left ventricular stroke volume was also higher for patients with raised PCWP (80 ± 26, *P* < 0.0001). Left ventricular ejection fraction did not differ significantly. Left ventricular mass (LVM) and LAV were higher in those with a raised PCWP at 106 ± 38 g and 104 ± 51 cm^3^ (*P* < 0.0001), respectively (*Figure 1* and *Structured Graphical Abstract*). Volumetric assessment of the right heart showed patients with raised PCWP to have a higher RVEDV (160 ± 62 mL, *P* < 0.0001) and RVESV (90 ± 48 mL, *P* = 0.024). Right ventricular ejection fraction did not differ between patients with normal and raised PCWP.

### Derivation cohort

A total of 708 (85%) patients were included in the derivation cohort (*Table 2*). Left atrial volume was most strongly associated with RHC PCWP (*r* = 0.55, 95% CI: 0.50–0.61, *P* < 0.0001) (*Table 3*). Left ventricular end-diastolic volume and LVESV had an association of 0.32 (*P* < 0.0001) and 0.23 (*P* < 0.0001), respectively. Left ventricular mass was moderately associated (*r* = 0.28, *P* < 0.0001). Right heart metrics were less strongly associated; RVSV had an *r*-value of 0.23 (*P* < 0.0001), RVEDV (*r* = 0.14, *P* < 0.0003) and RVEF of 0.08 (*P* = 0.03). Right ventricular end-systolic volume was not associated (*r* = 0.05, *P* = 0.21). At backward multivariate regression, two CMR variables showed an independent association with invasively measured PCWP; LAV and LVM. The following equation was derived:

$$\mathrm{CMR}\;\mathrm{PCWP}=6.1352+(0.07204{\;}^{*}\;\mathrm{LAV})+(0.02256{\;}^{*}\;\mathrm{LVM}).$$

**Table 2:** Patient characteristics, cardiac haemodynamic data and cardiac magnetic resonance data stratified by derivation and validation cohort

	Derivation cohort (*n* = 708)	Validation cohort (*n* = 127)	*P*-value
Age (years)	66.3 ± 13.2	66.0 ± 12.7	0.82
Male sex	295 (42%)	43 (34%)	0.10
Body surface area (m^2^)	1.91 ± 0.25	1.87 ± 0.22	0.22
HFpEF	371 (52%)	71 (56%)	0.47
HFmEF	29 (4.1%)	3 (2.4%)	0.34
HFrEF	15 (2.1%)	8 (6.3%)	0.008
Other	293 (41%)	45 (35%)	0.20
Heart rate (bpm)	76.0 ± 14.2	75.2 ± 14.7	0.5670
Systolic blood pressure (mmHg)	143.0 ± 26.2	145.6 ± 30.3	0.3174
Diastolic blood pressure (mmHg)	77.5 ± 12.6	78.9 ± 14.0	0.2644
Mean arterial pressure (mmHg)	102.0 ± 17.5	103.9 ± 18.9	0.2854
Mean PCWP (mmHg)	14.0 ± 6.2	13.4 ± 6.3	0.2866
Mean right atrial pressure (mmHg)	10.2 ± 5.8	8.5 ± 5.2	0.0016
Mean pulmonary artery pressure (mmHg)	38.4 ± 13.7	35.4 ± 14.0	0.0265
Systolic pulmonary artery pressure (mmHg)	63.3 ± 24.1	58.3 ± 23.2	0.0317
Diastolic pulmonary artery pressure (mmHg)	22.2 ± 9.6	20.1 ± 10.1	0.0230
Arterial oxygen saturations (%)	94.0 ± 4.3	95.4 ± 3.5	0.0006
Venous oxygen saturations (%)	65.7 ± 8.4	67.2 ± 9,1	0.0866
Cardiac output (L)	5.0 ± 2.0	4.9 ± 1.5	0.5523
Cardiac index (L/min/m^2^)	2.7 ± 1.0	2.7 ± 0.8	0.8798
Left ventricular end-diastolic volume (mL)	110.4 ± 37.7	107.8 ± 34.7	0.4551
Left ventricular end-systolic volume (mL)	37.4 ± 20.6	37.9 ± 25.9	0.8106
Left ventricular stroke volume (mL)	73.21 ± 24.7	69.9 ± 23.2	0.1587
Left ventricular ejection fraction (%)	66.9 ± 10.7	66.2 ± 13.7	0.5272
Left ventricular mass (g)	96.6 ± 32.1	105.4 ± 41.1	0.007
Right ventricular end-diastolic volume (mL)	149.3 ± 62.5	138.2 ± 58.0	0.0617
Right ventricular end-systolic volume (mL)	86.8 ± 49.8	76.3 ± 44.7	0.0275
Right ventricular stroke volume (mL)	62.6 ± 26.7	61.8 ± 26.0	0.7679
Right ventricular ejection fraction (%)	43.9 ± 13.5	46.5 ± 13.9	0.0454
Left atrial volume (cm^3^)	80.0 ± 43.8	72.5 ± 41.7	0.1239
Left ventricular end-diastolic volume (mL) (indexed)	58.2 ± 18.5	58.1 ± 19.1	0.9784
Left ventricular end-systolic volume (mL) (indexed)	19.6 ± 10.2	20.5 ± 14.9	0.3908
Left ventricular stroke volume (mL) (indexed)	38.6 ± 12.4	37.6 ± 12.4	0.3881
Left ventricular ejection fraction (%) (indexed)	50.6 ± 14.4	56.4 ± 21.4	0.0001
Left ventricular mass (g) (indexed)	78.8 ± 31.6	73.7 ± 29.1	0.0911
Right ventricular end-diastolic volume (mL) (indexed)	45.7 ± 25.6	40.5 ± 22.2	0.0319
Right ventricular end-systolic volume (mL) (indexed)	33.0 ± 13.7	33.2 ± 14.0	0.9438

**Table 3:** Univariate and multivariate linear regression comparing association between right heart catheterization pulmonary capillary wedge pressure and cardiac magnetic resonance pulmonary capillary wedge pressure

		Univariate		Multivariate^a^		
	(R)	Regression coefficient	*P*-value	Regression coefficient	SE	*P*-value
LV end-diastolic volume (mL)	0.32	0.05	<0.0001	—	—	—
LV end-systolic volume (mL)	0.23	0.07	<0.0001	—	—	—
LV stroke volume (mL)	0.29	0.07	<0.0001	—	—	—
LV ejection fraction (%)	−0.03	−0.02	0.40	—	—	—
LV mass (g)	0.28	0.05	<0.0001	0.023	0.006	0.0004
Left atrial volume (mL)	0.55	0.08	<0.0001	0.072	0.005	<0.0001
RV end-diastolic volume (mL)	0.14	0.02	0.0003	—	—	—
RV end-systolic volume (mL)	0.05	0.006	0.21	—	—	—
RV stroke volume (mL)	0.23	0.05	<0.0001	—	—	—
RV ejection fraction (%)	0.08	0.04	0.031	—	—	—
Constant		—	—	6.14	—	—
*R* ^2^		—	—	0.31	—	—
Multiple correlation coefficient		—	—	0.56	—	—

a Backward regression.

*—* represents *P* < 0.0001. R, Pearson correlation; SE, standard error of regression coefficient; LV, left ventricular; RV, right ventricular; R^2^ = coefficient of determination.

The model was not improved with the use of indexed parameters (Supplementary material online, *Table S2*) [R = 0.56 (non-indexed) vs. 0.52 (indexed)] nor with the use of more advanced machine learning techniques (Supplementary material online, *Table S1*).

### Validation cohort

The above equation was applied to the validation cohort (*n* = 127) to predict PCWP. Both RHC- and CMR-derived PCWP were comparable (13.4 ± 6.3 vs. 13.7 ± 3.1, *P* = 0.43). The correlation coefficient between RHC PCWP and CMR-modelled PCWP was 0.55 (95% CI: 0.41–0.66, *P* < 0.0001). When the CMR-modelled PCWP was tested in different sub-phenotypes of HF, it demonstrated a good association to invasively measured PCWP. The correlation was strongest in patients with HF with mid-range ejection fraction (HFmrEF) (*r* = 0.99, *P* < 0.001). Associations of 0.68 (*P* < 0.001) and 0.45 (*P* < 0.001) were found in HFrEF and HFpEF, respectively. The diagnostic accuracy of CMR-derived PCWP to predict elevated LVFP (PCWP ≥15 mmHg) was 76% (sensitivity 39%, specificity 92%, positive predictive value 66%, negative predictive value 78%). The area under the curve was 0.81 (95% CI: 0.74–0.89) (Supplementary material online, *Figure S1*, *P* < 0.001). Even the bias between the RHC- and CMR-derived PCWP was minimal (bias = –0.37 mmHg, 95% CI: –1.3 mmHg to 0.56 mmHg, *P* = 0.43) (Supplementary material online, *Figure S2*). The diagnostic accuracy in HFrEF, HFmrEF, and HFpEF was 100%, 100%, and 61%, respectively.

### Comparison with transthoracic echocardiography

All 127 patients in the validation cohort underwent TTE within 24 h of RHC. Based on the ASE algorithm, LAP was classified as raised in 18 (14%), normal in 47 (37%) and indeterminate in 62 (49%) cases. The results of the TTE assessment were concordant with RHC PCWP in 32 (25%) cases. Of those where TTE was non-diagnostic (indeterminate or incorrect diagnosis), CMR correctly reclassified to normal or raised LAP in 67 (71%) cases.

### Survival analysis

**Figure 2:**
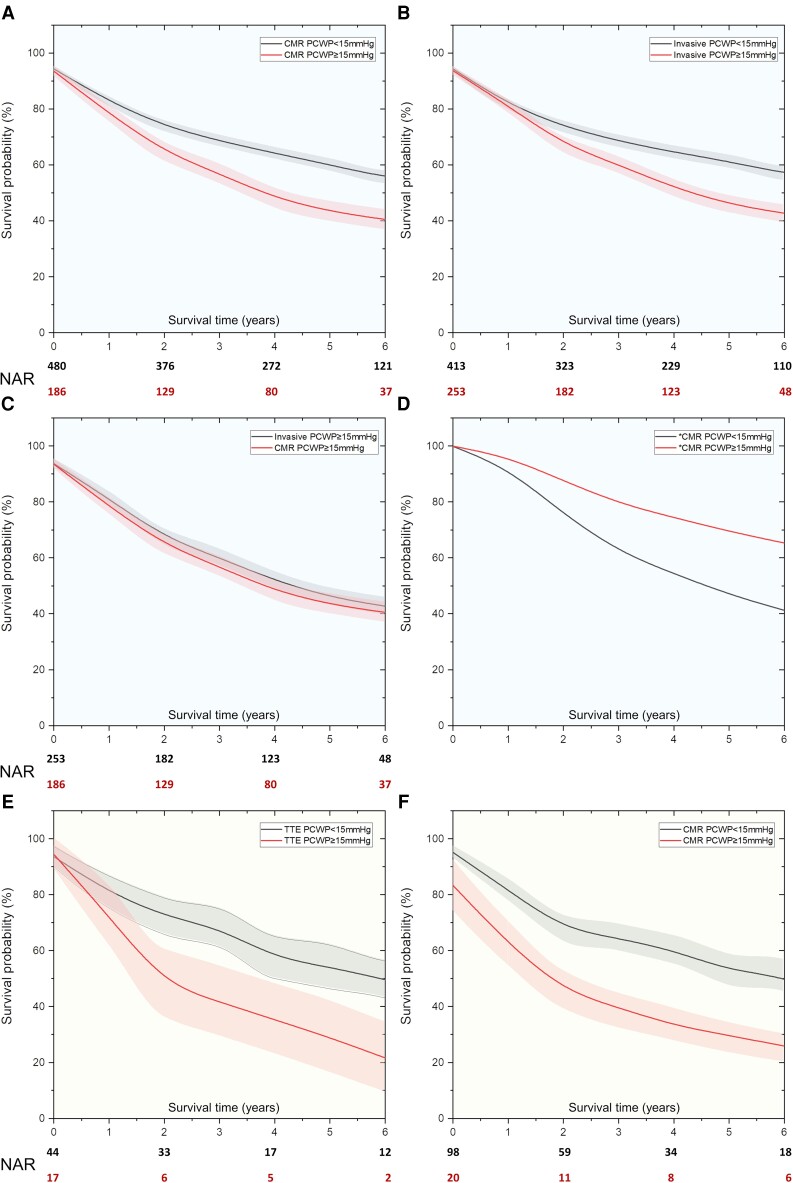
(*A*,*B*) Kaplan–Meier survival curves demonstrate prognostic relevance of both invasive and non-invasive pulmonary capillary wedge pressure. (*C*) Cardiac magnetic resonance-derived pulmonary capillary wedge pressure was non-inferior to an invasively measured pulmonary capillary wedge pressure. (*D*) After adjusting for all cardiac magnetic resonance variables associated with mortality, cardiac magnetic resonance-modelled pulmonary capillary wedge pressure independently predicted outcomes. (*E,F*) In the validation cohort, the transthoracic echocardiography and cardiac magnetic resonance models both independently predicted outcomes. Early outcomes were better predicted with the cardiac magnetic resonance model. CMR, cardiac magnetic resonance; PCWP, pulmonary capillary wedge pressure; TTE, transthoracic echocardiography; RHC, right heart catheterization; NAR, Numbers at risk.

Both RHC PCWP and CMR-modelled PCWP could predict survival over a mean follow-up of 5.2 ± 0.3 years (χ^2^ = 13.2, *P* < 0.001, and χ^2^ = 15.7 *P* < 0.001, respectively) (*Figure 2*). Both RHC-measured and CMR-modelled PCWP (≥15 mmHg) were comparable to predict survival at a maximum follow-up duration of 7 years (χ^2^ = 0.41, *P* = 0.52). In univariate Cox proportional hazards regression (*Table 4*), CMR-modelled PCWP (HR: 1.77, *P* = 0.04) demonstrated association with mortality. Right heart catheterization and TTE PCWP were non-significant. After adjusting for all the CMR parameters (LVEDV, LVESV, LVSV, LVM, and LAV), CMR PCWP ≥15 mmHg still was associated with poor survival (25 vs. 54% at 7-year follow-up, χ^2^ = 5.0, *P* = 0.03).

**Table 4:** Cox proportional hazards regression in the validation cohort

Comparing CMR variables	Regression coefficient	SE	HR	95% CI	*P*-value
Univariate Cox proportional hazards regression					
CMR PCWP	0.105	0.03	1.11	1.04–1.19	0.002
LA volume	0.008	0.00	1.01	1–1.01	0.002
Multivariate Cox proportional hazards regression					
LV mass	0.003	0.00	1.00	0.99–1.01	0.27
CMR PCWP	0.105	0.03	1.11	1.04–1.19	0.002
Comparing imaging methods					
Invasive PCWP ≥15 mmHg	0.253	0.25	1.29	0.79–2.11	0.32
CMR PCWP ≥15 mmHg	0.573	0.28	1.77	1.03–3.06	0.04
TTE PCWP ≥15 mmHg	0.583	0.36	1.79	0.88–3.64	0.11

a Standardized using *Z*-scores. SE, standard error of regression coefficient; HR, hazard ratio; CI, confidence interval; RHC, right heart catheterization; PCWP, pulmonary capillary wedge pressure; CMR, cardiac magnetic resonance; TTE, transthoracic echocardiography; LA, left atrium; LV, left ventricular.

## Discussion

The present study demonstrated that in patients with suspected HF, CMR volumetric variables can be used to predict elevated LVFP, significantly improving the classification provided by standard TTE assessment. Furthermore, the rise in CMR-modelled PCWP was associated with an increased risk of death. Notably, the prognostic power of CMR-modelled PCWP was non-inferior to RHC-measured PCWP.

Any rise in intracardiac pressure due to cardiac insufficiency results in remodelling of both the atrium and ventricle. In this study, we noted that invasively measured PCWP, a surrogate of LVFP, had a positive association with CMR-derived LAV and LVM. These findings are consistent with the Frank–Starling mechanism underpinning cardiovascular physiology, i.e. ventricular output increases as pre-load (end-diastolic pressure) increases.^15–17^

Left ventricular diastolic dysfunction is associated with LV hypertrophy (LVH), which can be quantified using LVM.^18,19^ Moreover, LVH is independently associated with a poorer prognosis.^20–22^ As expected, this study, which recruited a large heterogeneous cohort of patients, confirms that an increase in LVM is associated with LVFP. Left atrial remodelling known to result from the cumulative effect of raised LVFP in chronic HF^8^ was also associated with RHC-measured PCWP.

These two main structural pathophysiological changes due to raised LVFP, namely dilated LA (predominantly pre-load related) and LV hypertrophy (predominantly afterload related), form the basis of the American College of Cardiology and American Heart Association grading system, commonly used to assess suspected HF patients, and are crucial components of a true physiological model. Excellent spatial resolution and unrestricted field of view in CMR offer an improved assessment of structural abnormalities in comparison with echocardiography and also enhance functional assessment in patients with structural abnormalities.^23^

The CMR model includes only parameters fundamental to the pathophysiology of HF. It highlights the key metrics common to all HF patients and is thus simple to translate into clinical practice. Whilst RHC has long been the gold standard for HF assessment, its coefficient of variability remains high, partly due to the technical expertise needed.^24^ In contrast, CMR may offer a non-invasive estimate of LVFP at a reduced cost and with similar, if not improved, prognostic power.^25^ Moreover, the physiological CMR model allows for results to be interpreted easily and quickly. On Cox proportional hazards regression, our model was superior to PCWP in predicting mortality. This may in part reflect the relatively high coefficient of variation seen when measuring PCWP on RHC. Furthermore, the CMR model demonstrated a degree of proportional bias with a tendency to overestimate at higher PCWP values. Finally, the coefficient of determination (R-squared) of the CMR model was 0.31. Therefore, although the model provides excellent diagnostic accuracy, only 31% of the variation in PCWP is accounted for by the model. These discrepancies could explain the differing relationship with outcomes between RHC PCWP and CMR-modelled PCWP.

Transthoracic echocardiography-based estimations of LVFP are mainly carried out by measuring trans-mitral inflow and mitral annular diastolic velocities. These are incorporated in the ASE guidelines for the assessment of diastolic dysfunction.^8^ However, these techniques provide a largely dichotomized assessment limiting flexible clinical use. In the present study, we demonstrated that the CMR model has a complementary role to a standard TTE-guided algorithm for the prediction of raised filling pressures. Based upon the TTE algorithm, LAP was correctly classified in just 25% of patients and was indeterminate in 49%. Of those where TTE was non-diagnostic (indeterminate or incorrect diagnosis), CMR correctly reclassified patients to normal or raised LAP in 67 (71%). Early validation studies of this TTE-guided approach report a diagnostic accuracy in the region of 75–85%,^26–28^ far superior to that reported in the present study. However, when applied to patients with preserved ejection fraction alone, van de Bovenkamp *et al*. demonstrated a significant reduction of accuracy to 67% with a sensitivity of 35%. In addition, 30% of cases produced an indeterminate result.^29^ We observed a similarly high level of indeterminate results.

**Figure 3:**
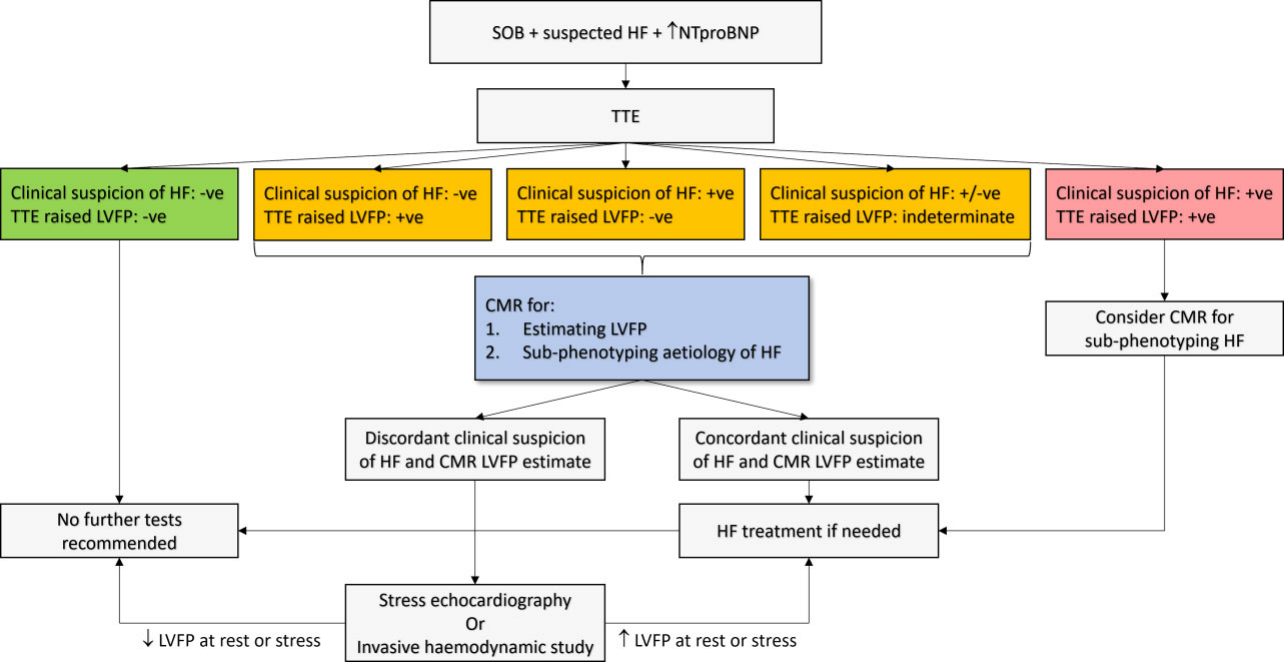
Proposed decision-making algorithm for the assessment of raised left ventricular filling pressure. -ve, negative; +ve, positive; CMR, cardiovascular magnetic resonance; LVFP, left ventricular filling pressure; SOB, shortness of breath; HF, heart failure; TTE, transthoracic echocardiography

Our study consists of a heterogeneous population and is the largest of its kind. Many previous studies included small sample sizes in well-defined clinical groups, possibly explaining the superior performance of early TTE models.^30^ In addition, this was a real-world study using standard TTE acquired for clinical use. In routine clinical practice, TTE is performed by a wide variety of operators with differing experiences and skill. There is therefore significant variation in the parameters obtained and accuracy of the measurements taken which may negatively impact LAP estimation. Conversely, the relevant CMR parameters can be obtained using standard protocols, with high repeatability. There is therefore likely to be less variability, and therefore higher reliability, with CMR-based models compared with TTE. Moreover, we speculate that this CMR-informed model may be less susceptible to pre-loading conditions as it incorporates physiological parameters, particularly LVM, which is less susceptible to immediate changes in loading conditions.^31^ Left atrial volume may be more susceptible to pre-loading conditions^32^; however, the value of such parameters is in making the model more dynamic. This becomes more relevant if CMR studies are going to be used to estimate LVFP in the same patient immediately pre- and post-treatment. These characteristics potentially make the novel CMR-modelled PCWP proposed in this study, a more clinically useful non-invasive imaging-based surrogate of LVFP in both routine practices and at follow-up, particularly in assessing therapeutic response, and in reducing the need for invasive testing. Cardiac magnetic resonance remains the gold standard for volumetric and functional assessment and in the classification of HF aetiology. It is also important to note that prognostication is an important aspect of any diagnostic test. The results of this study demonstrate that CMR-informed LVFP is non-inferior to invasive LVFP for informing prognosis. Other CMR studies have also echoed CMR’s prognostic role in the assessment of HF.^33,34^ Therefore, many patients with suspected HF will undergo clinically indicated CMR, making our model widely applicable. Where this is not the case, CMR could be reserved for cases where TTE assessment is indeterminate or clinical suspicion is high despite a normal LAP as determined by TTE. In patients with HFpEF, TTE-based models of LAP perform less well,^30^ and CMR with LVFP prediction, may be considered earlier in the diagnostic algorithm recently proposed by the consensus paper,^35^ where the diagnosis is unclear (*Figure 3*).

### Future work

In this study, we developed a volumetric-based CMR model to predict invasively measured PCWP. This CMR LVFP model can easily be incorporated into routine clinical workflows. Volumetric and functional assessment by CMR is rapidly moving from manual segmentation, which takes considerable time and effort, to automated segmentation using artificial intelligence.^36,37^ Our proposed CMR-modelled PCWP can be generated almost instantaneously once the CMR-derived LV and LA volumetric assessments have been made. Our study did not incorporate any tissue characterization parameters by CMR. Myocardial tissue mapping techniques including T1-/T2-mapping and late gadolinium enhancement imaging may allow further improvement in the precision of the model by factoring in myocardial fibrosis or scar.^38^ In particular, the extracellular volume has shown promise and is likely to add incremental value to our model.^39,40^ Novel emerging CMR technologies including four-dimensional flow mapping are likely to further enhance the model.^41–45^ More recent echocardiographic advanced models using LA strain have demonstrated better accuracy for LVFP estimation, especially considering patients with preserved ejection fraction. Left atrial strain assessment does not have a similar limitation to Doppler as it is not angle independent. More recently, an emerging role of these advanced echocardiographic indices to estimate LVFP has been recognized by a consensus paper on the role of multi-modality imaging in HFpEF.^35^ Hence, future studies are warranted to investigate not only the emerging complementary role of CMR to estimate LVFP but also the incremental role of advanced echocardiographic methods in routine estimation of LVFP.

### Limitations

This was a single-centre observational study. There is a possibility of selection bias as this study was performed at a tertiary centre that took referral for RHC assessment. This may be the reason why the mean pulmonary artery pressure in the whole population was elevated. Despite that, this is one of the largest studies that recruited a heterogeneous cohort of patients to investigate LVFP by CMR. Importantly, all patients recruited to this study were clinically stable and represent real-world patients presenting with shortness of breath to outpatient departments. In this study, we did not assess any acute HF patients needing intravenous therapy and, hence, the results from this study cannot be applied to acutely decompensated HF patients. Bland–Altman analysis demonstrated a degree of proportional bias (Supplementary material online, *Figure S2*) with a tendency to underestimate at lower PCWP values and overestimate at higher PCWP values. However, despite this, the diagnostic accuracy remains good, with superiority over TTE-derived estimates. The primary purpose of this model is to correctly categorize patients into normal or raised LVFP, which our model achieves. More work will be required to determine if there is a role for more accurate quantification of PCWP for risk stratification and monitoring purposes. This will be the focus of future studies. As this was a retrospective study in a specified population, the value of the proposed algorithm in non-selected patients recruited prospectively remains to be tested. Transthoracic echocardiography was only analysed in patients in the validation cohort to allow comparison with CMR-derived estimates of LVFP. We are therefore unable to comment on the performance of TTE-based algorithms in a larger cohort. Furthermore, the TTEs analysed in this study were performed as part of routine clinical practice according to BSE guidance. Advanced echo techniques such as the analysis of LV/LA global longitudinal strain and pulmonary vein flow are likely to significantly improve the prediction of LVFP but were not performed in this study. Therefore, we cannot comment on a comparison between our CMR model and these techniques.

## Conclusions

A physiological CMR model consisting of volumetric and functional metrics can accurately predict invasively measured PCWP and improves classification provided by standard TTE models. Furthermore, the CMR-modelled PCWP has a prognostic role that is non-inferior to RHC-measured PCWP.

## Supplementary material

Supplementary material is available at the *European Heart Journal* online.

## Declaration of Helsinki

This study complies with the Declaration of Helsinki and was approved by the National Research Ethics Service (16/YH/0352) in the UK.

## Supplementary Material

ehac207_Supplementary_Data

## Data Availability

The data underlying this article will be shared on reasonable request to the corresponding author.
