# Supplementary material for: Cardiac magnetic resonance identifies raised left ventricular filling pressure: prognostic implications
Source: Eur Heart J. 2022 May 4;43(26):2511–22. doi: 10.1093/eurheartj/ehac207 (PMC9259376; doi:10.1093/eurheartj/ehac207)

## Supplementary Online Document

**S Figure 1**: Receiver operating characteristic curve for CMR modelled PCWP.

ROC curve for CMR modelled PCWP with comparison against measured PCWP > 15mmHg to identify raised left ventricular filling pressures. Area under the curve (AUC) = 0.81 (95% CI 0.74 to 0.89).


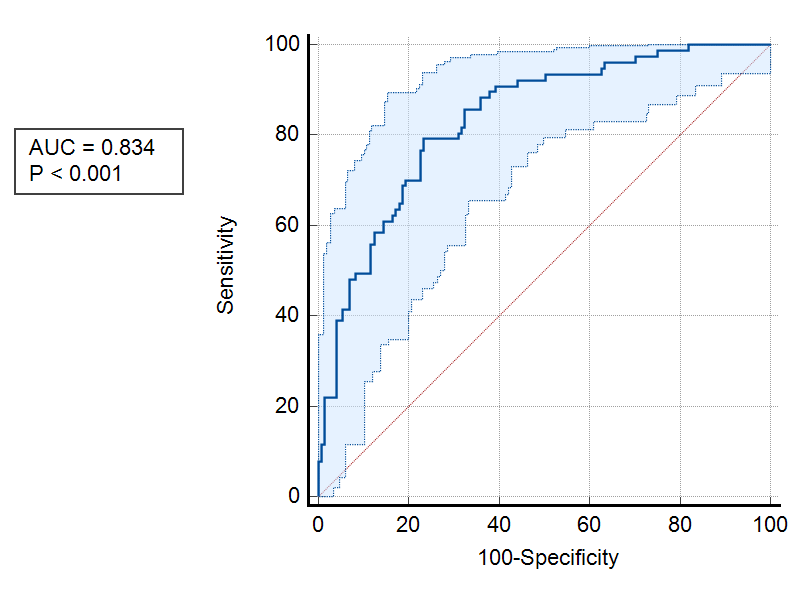


**S Figure 2.** Bland-Alman plot of the measured versus modelled PCWP in the validation cohort (n=127).


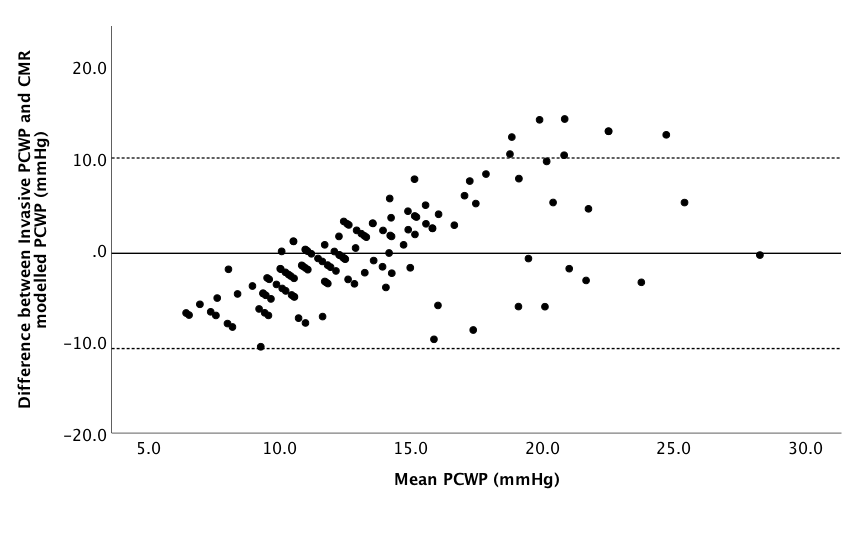

Supplement: ehac207_Supplementary_Data [file ehac207_supplementary_data.zip › Supplementary Online Document.docx]
