# Supplementary material for: Cardiac magnetic resonance identifies raised left ventricular filling pressure: prognostic implications
Source: Eur Heart J. 2022 May 4;43(26):2511–22. doi: 10.1093/eurheartj/ehac207 (PMC9259376; doi:10.1093/eurheartj/ehac207)
Supplement: ehac207_Supplementary_Data [file ehac207_supplementary_data.zip › Supplementary tables.docx]

**Supplementary tables**

Supplementary table 1: A comparison of CMR based models of invasive PCWP derived using multiple linear regression and machine learning techniques.

|  | **Multiple linear regression** | **Machine learning techniques** | | |
| --- | --- | --- | --- | --- |
|  | **Linear regression (Stepwise)** | **Elastic Net** | **Lasso** | **Lasso (adaptive)** |
| **LV end diastolic volume (ml)** |  | 0.008 | 0.008 | 0.02 |
| **LV end systolic volume (ml)** |  | 0.0006 | 0.0005 |  |
| **LV mass (g)** | 0.02 | 0.01 | 0.01 | 0.0008 |
| **LA volume (cm^3^)** | 0.07 | 0.07 | 0.07 | 0.07 |
| **LV stroke volume (ml)** |  | 0.00009 |  |  |
| **Constant** | 6.14 | 6.57 | 6.57 | 6.52 |
| **R-squared** | 0.31 | 0.31 | 0.31 | 0.31 |

Values are estimated regression coefficients. LV = left ventricle, LA = left atrium. R squared = coefficient of determination.

Supplementary table 2: A comparison of CMR derived of invasive PCWP using non-indexed and indexed LV and RV parameters.

LA = Left atrial, LV = left ventricular, EDV = end-diastolic volume, ESV = end-systolic volume, EF = ejection fraction, SV = stroke volume, RV = right ventricular. SE = standard error of the estimated regression coefficients.

| **Non-indexed LV and RV parameters** | | | |  | **Indexed LV and RV parameters** | | | |
| --- | --- | --- | --- | --- | --- | --- | --- | --- |
| **Sample size** | 708 |  |  |  | **Sample size** | 708 |  |  |
| **R^2^-adjusted** | 0.31 |  |  |  | **R^2^-adjusted** | 0.27 |  |  |
| **Correlation coefficient** | 0.56 |  |  |  | **Correlation coefficient** | 0.52 |  |  |
|  |  |  |  |  |  |  |  |  |
| **Regression Equation** |  |  |  |  | **Regression Equation** |  |  |  |
| **Independent variables** | **Correlation**  **coefficient** | **SE** | **P** |  | **Independent variables** | **Correlation**  **coefficient** | **SE** | **P** |
| **(Constant)** | 6.14 |  |  |  | **(Constant)** | 5.85 |  |  |
| **LA volume** | 0.07 | 0.00 | <0.0001 |  | **LA volume (indexed)** | 0.12 | 0.01 | <0.0001 |
| **LV mass** | 0.02 | 0.01 | 0.0004 |  | **LV mass (indexed)** | 0.06 | 0.01 | 0.0001 |
|  |  |  |  |  |  |  |  |  |
| **Variables excluded** |  |  |  |  | **Variables excluded** |  |  |  |
| LVEDV |  |  |  |  | iLVEDV |  |  |  |
| LVESV |  |  |  |  | iLVESV |  |  |  |
| LVSV |  |  |  |  | iLVSV |  |  |  |
| LVEF |  |  |  |  | LVEF |  |  |  |
| RVEDV |  |  |  |  | iRVEDV |  |  |  |
| RVESV |  |  |  |  | iRVESV |  |  |  |
| RVSV |  |  |  |  | iRVSV |  |  |  |
| RVEF |  |  |  |  | RVEF |  |  |  |
